# Persistent detection of dengue virus RNA in vaginal secretion of a woman returning from Sri Lanka to Italy, April 2017

**DOI:** 10.2807/1560-7917.ES.2017.22.34.30600

**Published:** 2017-08-24

**Authors:** Marco Iannetta, Eleonora Lalle, Maria Musso, Fabrizio Carletti, Laura Scorzolini, Alessandra D’Abramo, Pierangelo Chinello, Concetta Castilletti, Giuseppe Ippolito, Maria R Capobianchi, Emanuele Nicastri

**Affiliations:** 1 National Institute for Infectious Diseases ‘Lazzaro Spallanzani’, IRCCS, Rome, Italy

**Keywords:** Dengue, Zika, vaginal secretion, vaginal shedding, vertical transmission, sexual transmission, flavivirus

## Abstract

We describe the dynamics of dengue virus (DENV) infection in a woman in her mid-30s who developed fever after returning from Sri Lanka to Italy in April 2017. Laboratory testing demonstrated detectable DENV-RNA in plasma, urine, saliva, vaginal secretion. Persistent shedding of DENV-RNA was demonstrated in vaginal secretion, and DENV-RNA was detectable in the pelleted fraction up to 18 days from symptom onset. These findings give new insights into DENV vaginal shedding and vertical transmission.

We present a case of primary dengue fever (DF) in a Caucasian woman returning from Sri Lanka to Italy in April 2017. Dengue virus (DENV) RNA was persistently detected in vaginal secretion up to 18 days from symptom onset (FSO).

## Case report

In April 2017, a Caucasian woman in her mid-30s returning to Italy from a 19-day travel in Sri Lanka, experienced a 3-day course of fever (> 38.5 °C), arthralgia, weakness and headache. On admission at the National Institute for Infectious Diseases Lazzaro Spallanzani in Rome, Italy (day 3 FSO), a commercial dengue rapid test (Dengue DUO, Standard Diagnostics Inc., Kyonggi-do, Korea), detecting specific IgG, IgM and non-structural (NS)-1 protein, revealed NS-1 antigen reactivity only. Routine laboratory tests showed transient leukopenia and thrombocytopenia, with slight increase of liver enzymes and alterations of coagulation parameters (Figure).

**Figure:**
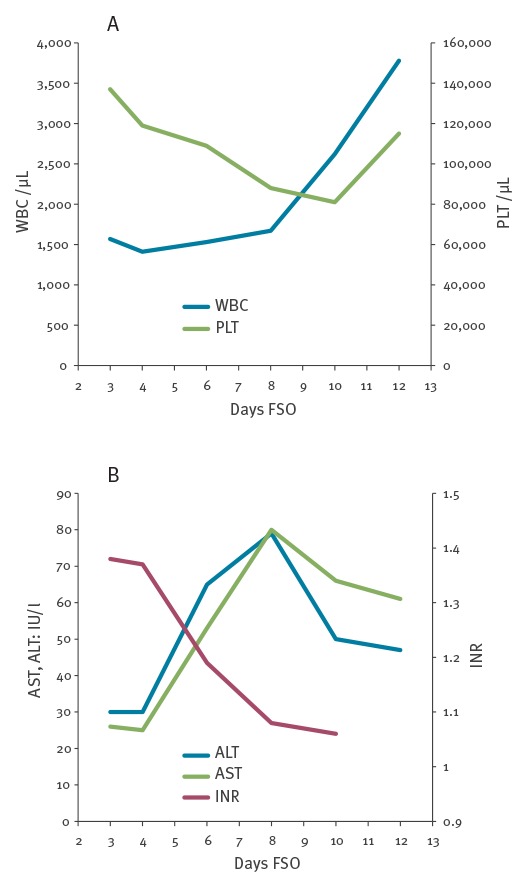
Laboratory tests performed during hospitalisation, case of dengue fever, Italy, April 2017

FSO: from symptom onset.

A: white blood cell (WBC) and platelet (PLT) absolute counts.

B: liver enzymes alanine aminotransferase (ALT), aspartate aminotransferase (AST), international normalised ratio (INR).

On day 4 FSO, DENV-specific IgG and IgM, assessed by indirect immune fluorescence assay (IFA, Arboviral Fever Mosaic-2, IgM and IgG, Euroimmun, Hamburg, Germany), were below the detection threshold (1/20), and real-time PCR for DENV (CDC DENV-1–4 Real-Time RT-PCR Assay, Atlanta, United States (US)) was positive in serum (cycle threshold (C_t_): 22); hence the diagnosis was primary DENV infection. Viral RNA was also detected in urine (C_t_: 34.61), saliva (C_t_: 33.55) and vaginal secretion (C_t_: 30.71). The latter was collected by flocked swab and immediately suspended in 2 mL of vaginal swab transport medium (VSTM) at 4 °C in a 15-mL sterile tube. Moreover, a pan-flavivirus genus-specific nested RT-PCR targeting the NS-5 gene (modified from [1]) followed by the amplicon sequencing, showed DENV type 2 in all the collected samples (saliva, urine, serum and vaginal swab). The virological investigation for DENV was repeated on longitudinally collected samples (Table).

**Table:** Dengue virus-RNA detection and serology at different time points, case of dengue fever, Italy, April 2017

Days FSO	DENV-RNA RT-PCR						DENV serology		
	Serum	Urine	Saliva	Vaginal swab			IgM	IgG	NS1
				Total VSTM	PeF	SNF			
**3**	NA	NA	NA	NA	NA	NA	Neg^a^	Neg^a^	Pos^a^
**4**	Pos (Ct: 22.00)	Pos (Ct: 34.61)	Pos (Ct: 33.55)	Pos (Ct: 30.71)	NA	NA	< 1:20	< 1:20	NA
**10**	Pos (Ct: 32.96)	Pos (Ct: 31.50)	Pos (Ct: 37.29)	Pos (Ct: 35.47)	Pos (Ct: 35.47)	Pos (Ct: 33.34)	1:20	< 1:20	NA
**18**	Neg	Pos (Ct: 32.53)	Neg	Neg	Pos (Ct: 35.89)	Neg	1:80	1:160	NA
**36**	Neg	Neg	Neg	Neg	Neg	Neg	1:160	1:320	NA

^a^ Rapid test.

Ct: cycle threshold; DENV: dengue virus; FSO: from symptom onset; NA: not available; Neg: negative; PeF: pelleted fraction; Pos: positive; SNF: supernatant fraction; VSTM: vaginal swab transport medium.

Rapid test, thin and thick smear and PCR for malaria were all negative; chikungunya and Zika virus serologies were negative as well as specific PCR in serum and urine. Epstein-Barr virus and Cytomegalovirus serology indicated past infections, with negative IgM for both viruses.

On day 10 FSO, serum, urine, saliva, and vaginal swab were collected, and DENV-RNA was still detectable in all the samples (serum C_t_: 32.96; urine C_t_: 31.50; saliva C_t_: 37.29; VSTM C_t_: 35.47). VSTM was centrifuged at 1,500 rpm for 10 minutes to obtain supernatant fractions (SNF) and pelleted fractions (PeF). Total RNA was extracted from SNF and PeF using the COBAS AmpliPrep Total Nucleic Acid Isolation Kit (Roche, Indianapolis, Indiana, US) and Trizol (Life Technologies, Stockholm, Sweden) respectively, according to the manufacturer’s instructions. RT-PCR for DENV-RNA resulted positive in SNF and PeF with a C_t_ of 35.47 and 33.34, respectively.

On day 18 FSO, DENV-RNA was detectable only in urine (C_t_: 32.53), while it was undetectable in serum, saliva and VSTM. After separation of VSTM, DENV-RNA was undetectable in SNF, while it was detected in PeF (C_t_: 35.89).

On day 36 FSO, DENV-RNA was no longer detectable in serum, urine, saliva, total VSTM and SNF and PeF (Table).

Specific IgM became detectable on day 10 FSO, and IgG and IgM titres rose on day 18 FSO (1/160 and 1/80, respectively) and on day 36 (1/320 and 1/160, respectively). Viral isolation from vaginal samples using VeroE6 cell culture was not successful.

## Background

DF is an arthropod-borne viral infection, transmitted to humans through mosquito bites and caused by DENV, a single stranded, positive-sense RNA virus, belonging to the genus Flavivirus, like Zika virus (ZIKV) and West Nile virus (WNV). Four serotypes (from 1 to 4) have been identified so far and each DENV serotype accounts for multiple phylogenetically-related genotypes [2]. DENV is widely diffused, causing more than 58 million symptomatic infections in 2013 [3]. Infection during pregnancy can cause severe maternal and neonatal complications [4,5], However, no fetal abnormalities have been reported so far after DENV infection in pregnant women. Vertical transmission has been described in several reports [6,7], whereas sexual transmission of DENV has never been reported.

## Discussion

The recent ZIKV outbreak evidenced the occurrence of vertical transmission of the virus with fetal abnormalities [8] and male-to-woman, woman-to-male and male-to-male sexual transmission [9], and highlighted the presence of infectious virus in almost all body secretions, including those from male and female genital tract [10-13].

Here we report on DENV-RNA detection in vaginal secretion of an acute case of primary DF. Moreover, we report the detection of viral RNA with the pellet fraction of vaginal secretion after centrifugation. This could reflect DENV association to vaginal epithelial cells, although association with other components of the pellet may not be ruled out. Although vaginal shedding of DENV was protracted up to 18 days FSO, we were not able to isolate replication competent virus from the different fractions derived from genital secretion, probably because of low viral loads detected in the vaginal swab, influence of sample pH and virus particles degradation. However, our findings could give a deeper insight in DENV sexual and vertical transmission.

Little is known about vertical transmission of DENV and a recent meta-analysis by Xiong et al. [14], failed to demonstrate that maternal DENV infection during pregnancy might increase the risk of premature birth, low birth weight, miscarriage and stillbirth. DENV has been identified in newborns after Caesarean section and the virus was isolated from umbilical cord blood, indicating the possibility of intrauterine acquisition of the infection; the presence of the virus in the vaginal mucosa, shown in our patient, is consistent with the possibility that DENV can be vertically transmitted also during vaginal delivery, similarly to genital herpes [15].

Interestingly, an in vitro study by Chan et al. [16] demonstrated the ability of DENV type 2 to replicate in human cell lines derived from the cervix (HeLa) while no viral growth was observed in experimental infection of placenta (JEG-3), endometrium (HOSE6–3), prostate (LNCaP) and testis (833KE) cell lines. Conversely, all the above-mentioned cell lines were susceptible to ZIKV [16].

Further investigations in individuals with acute DF are needed in order to understand the implications of DENV genital shedding on vertical and sexual transmission. Viral isolation and innovative molecular methods to detect DENV in the replicative phase represent essential steps in this process.
